# Effect of anteromedial portal location on femoral tunnel inclination, length, and location in hamstring autograft-based single-bundle anterior cruciate ligament reconstruction: a prospective study

**DOI:** 10.1186/s43019-023-00202-5

**Published:** 2023-11-27

**Authors:** Abdulaziz Z. Alomar, Baraa Baltow, Ismail AlMogbil

**Affiliations:** 1 Department of Orthopaedic Surgery, College of Medicine, King Saud University, P.O. BOX 7805, 11472 Riyadh, Saudi Arabia; 2 Department of Orthopaedic surgery, AlHada Armed Forces Hospital, Ministry of defense, Taif, Saudi Arabia; 3 Department of Surgery, Unaizah College of Medicine and Medical Sciences, Qassim University, Qassim, Saudi Arabia

**Keywords:** Anterior cruciate ligament reconstruction, Anteromedial portal, Far anteromedial portal, Femoral tunnel, Tunnel inclination, Tunnel length

## Abstract

**Background:**

Portal positioning in arthroscopic anterior cruciate ligament reconstruction is critical in facilitating the drilling of the femoral tunnel. However, the traditional approach has limitations. A modified inferior anteromedial portal was developed. Therefore, this study aims to compare the modified and conventional far anteromedial portals for femoral tunnel drilling, assessing factors such as tunnel length, inclination, iatrogenic chondral injury risk, and blowout.

**Material and methods:**

Patients scheduled for hamstring autograft-based anatomical single-bundle arthroscopic anterior cruciate ligament reconstruction were divided into two groups: modified and far anteromedial groups. Primary outcomes include differences in femoral tunnel length intraoperatively, tunnel inclination on anteroposterior radiographs, and exit location on lateral radiographs. Secondary outcomes encompass tunnel-related complications and reconstruction failures. To identify potential risk factors for shorter tunnel lengths and posterior exits, regression analysis was conducted.

**Results:**

Tunnel parameters of 234 patients were analyzed. In the modified portal group, femoral tunnel length and inclination were significantly higher, with tunnels exhibiting a more anterior exit position (*p* < 0.05). A higher body mass index exerted a negative influence on tunnel length and inclination. However, obese patients in the modified portal group had longer tunnels, increased inclination, and a lower risk of posterior exit. Only a few tunnel-related complications were observed in the far anteromedial group.

**Conclusion:**

The modified portal allowed better control of tunnel length and inclination, ensuring a nonposterior femoral tunnel exit, making it beneficial for obese patients.

## Background

Portal positioning in arthroscopic anterior cruciate ligament (ACL) reconstruction is essential in facilitating access to the anatomical footprint and the drilling of the femoral tunnel. The transtibial route has been conventionally employed for femoral tunnel drilling. However, this method is associated with various shortcomings, including limited access to the femoral ACL footprint, imprecise anterior entry of the femoral tunnel, and vertical tunnel drilling. These drawbacks result in reduced rotational stability and an increased pivot shift [1]. Several techniques have been introduced to address these challenges, such as the outside-in, retrograde drilling, flexible femoral tunnel reamers, and transportal (TP) techniques [2–4]. Among these, the TP technique stands out, offering improved access to the femoral ACL anatomical footprint and an oblique femoral tunnel [5, 6]. Furthermore, the tunnel is directed precisely to the femoral ACL anatomical footprint site, in contrast to the nonanatomical entry approach employed in the transtibial route. The resultant graft mimics the actual ACL orientation, providing better rotatory and anteroposterior (AP) stability [7, 8].

A standard anteromedial (AM) portal is positioned 1 cm medially from the patellar tendon and slightly distal to the patellar inferior pole [7]. However, this standard AM portal-based femoral tunnel drilling method presents certain challenges. These challenges include the short femoral tunnel and the risk of posterior wall blowout when there is a slight misalignment [5, 9, 10]. Complications associated with the use of standard AM portals could arise from imprecise placement and variations in the direction of tunnel drilling [9, 11]. Considering the risk of posterior tunnel exit and blowout, several studies have recommended the utilization of an accessory far AM (FAM) portal. This FAM portal is designed to align the tunnel more orthogonally to the anatomical footprint with a longer tunnel [9, 10, 12–14]. Various FAM portal locations have been proposed, with or without inferior positioning, ranging from 1 to 3 cm medially and 1 to 2.5 cm inferior to the standard AM portal [10, 12, 15]. However, the FAM technique is not risk free. Complications, such as iatrogenic injuries occur to the medial meniscus (MM) and articular surface of the medial femoral condyle (MFC). This also includes the potential for posterior tunnel blowout [5, 9, 16]. The use of low-profile reamers and protection sleeves/cannulas has been recommended to mitigate the risk of MFC chondral injury [17].

Most studies that have compared the standard AM and FAM portal techniques are based on cadaveric investigations [6, 10]. Moreover, no clinical studies have established the superiority of either technique [18].

In this study, a modification of the AM portal was devised. The modified inferior AM (MIAM) portal was positioned approximately 1 cm medial to the patellar tendon and as inferior as possible, allowing for an entry just lateral to the anterior horn of the medial meniscus. Therefore, this prospective cohort study aims to assess variations in the femoral tunnel orientation, tunnel length, iatrogenic MFC chondral injury risk, and blowout with the MIAM and FAM portal-based drilling techniques for single-bundle ACL reconstruction (ACLR). This study hypothesizes that the MIAM portal is superior to the FAM portal regarding femoral tunnel length, exit, obliquity, risk of lateral wall blowout, and injury to the MFC.

## Material and methods

### Patient recruitment

This single-center study spanned 3 years, including a minimum 2-year clinical follow-up period, to document any late tunnel-related complications.

The study was approved by the relevant institutional review board (no. E-21-6237). All patients aged 18–50 years who were scheduled for primary ACLR were invited to participate. Written informed consent was obtained from all the patients. Through sample size calculations, it was determined that a minimum of 146 knee joints were required (each group, *n* = 73). This was necessary to achieve a statistical power of 80%, a confidence interval of 95% (two sided), establish an equal sample size ratio of 1 between the two groups, and a significance level of 0.05 (OpenEpi, Version 3). A mean (standard deviation) tunnel inclination of 39.2° ± 2° and 40.2° ± 2.3° was used for the two comparative groups based on a study conducted by Erdem et al. [18] In this study, retaining a maximum number of recruited patients throughout the study period was prioritized.

### Inclusion and exclusion criteria

The inclusion criteria were patients aged 18–50 years who required primary single-bundle ACLR because of ACL tears and provided their consent to participate in the study. The exclusion criteria were patients with open physes around the knee joint, as observed on plain radiographs. Additionally, patients with multiligamentous injuries, surgical deferrals, or revision ACLR were excluded.

### Patient grouping

**Fig. 1:**
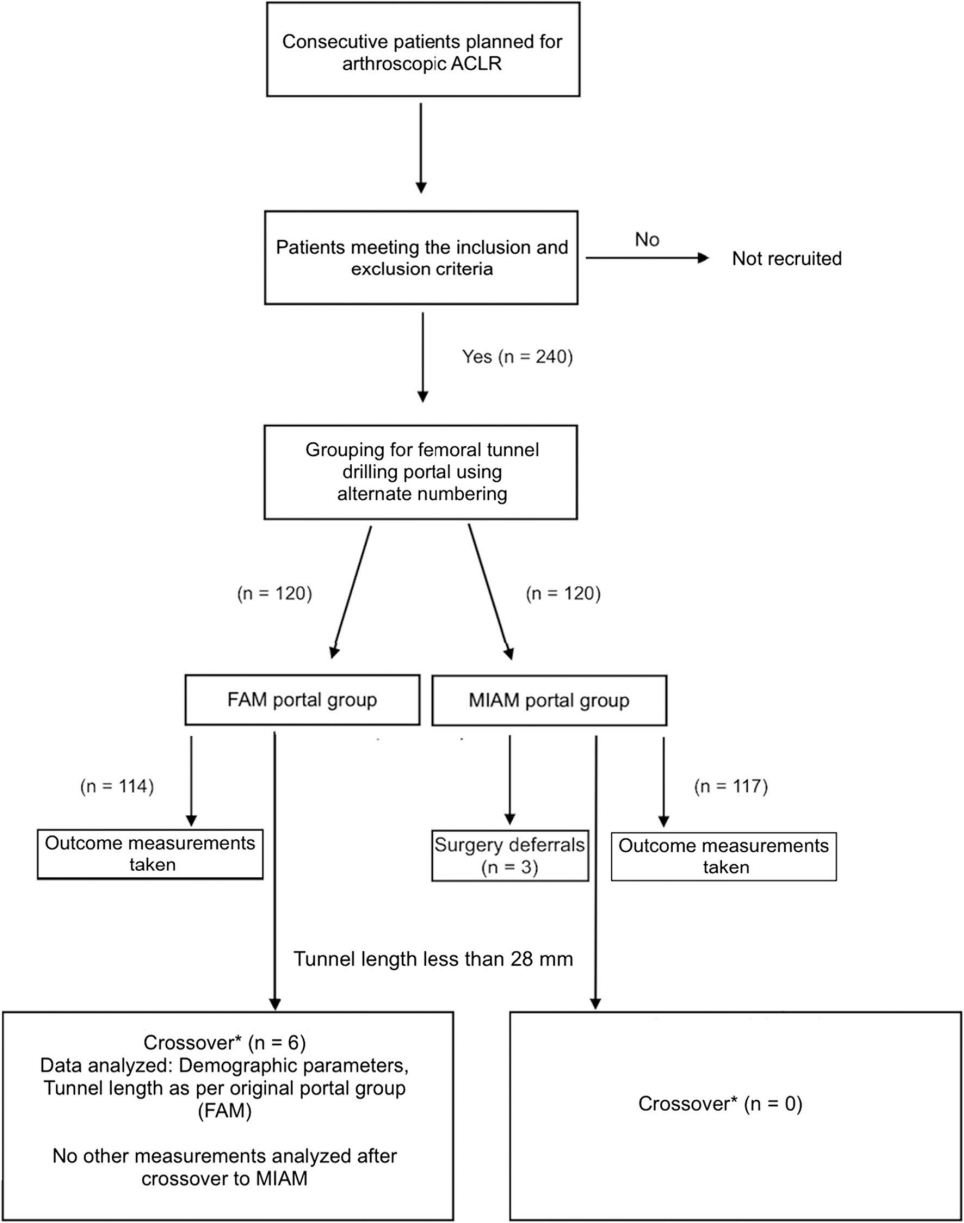
Flow diagram of patient recruitment and groupings based on FAM and MIAM portal techniques. *Crossover was performed exclusively for the portal technique. Six patients who underwent crossover contributed to the analysis of outcome measurements within the FAM group. This was undertaken before the change from a femoral tunnel-drilling portal to an MIAM portal. ACLR, anterior cruciate ligament reconstruction; FAM, far anteromedial; MIAM, modified inferior anteromedial

An investigator who was not part of the operating or data collection teams consecutively enrolled consenting patients alternately into two nonrandomized groups. These groups were based on two TP femoral tunnel drilling techniques, namely, the MIAM and FAM techniques. Special data entry personnel assigned unique identification code numbers to the enrolled patients and recorded the outcome measurement values of the study. These values were provided by the investigators, who were blinded to the surgical technique. Overall, 240 patients were recruited. However, three in the MIAM group were excluded because of surgical deferrals (Fig. 1).

### Surgical techniques

A single orthopedic surgeon, experienced in both techniques, performed all surgical procedures. For the hamstring autograft (gracilis and semitendinosus tendons), a closed fixed-loop suspensory fixation (Endobutton, Smith, and Nephew) was used and supported with a suspensory button on the femoral side. The portal techniques are described below.

#### The MIAM portal technique

**Fig. 2:**
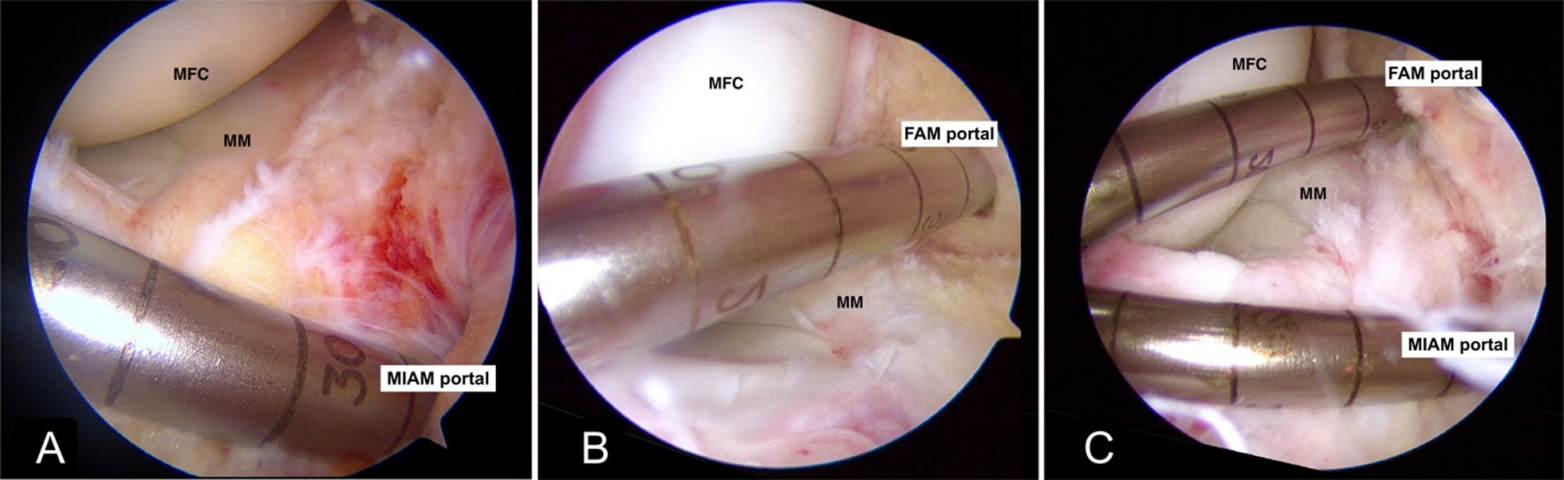
Intraoperative arthroscopic images showing portal orientation during the FAM and MIAM portal techniques. **A** An arthroscopic image of the right knee viewed through the anterolateral portal shows the meniscus-free zone in the MIAM portal for femoral tunnel drilling. **B** The FAM portal is located proximal to the medial femoral condyle at a more medial location. **C** A comparison illustrating the distinct drilling directions employed for femoral tunnel drilling via these portals. FAM, far anteromedial; MIAM, modified inferior anteromedial; MFC, medial femoral condyle; MM, medial meniscus

Following navigation through a standard anterolateral (AL) portal, the MIAM portal was performed under arthroscopic guidance. A spinal needle was inserted through the desired entry point, situated just lateral to the anterior horn of the MM, and positioned as inferior as possible, just superior to the anterior tibial rim. For inferior positioning of the portal, the anterosuperior edge of the tibial plateau was palpated, ensuring a direct entry above it. Subsequently, a vertical incision was created with the entry blade oriented in a superolateral direction, targeting the intercondylar notch, thereby preventing anterior inter-meniscal ligament injury (Fig. 2). The MIAM portal was also used to prepare the femoral and tibial footprints of the torn ACL and address concomitant procedures, such as meniscus repair, eliminating the need for an additional AM portal creation.

#### The FAM portal technique

The FAM portal was created under arthroscopic guidance with the aid of a spinal needle. The needle was carefully positioned at the farthest medial location, ensuring its trajectory towards the ACL footprint while remaining clear of the MFC and the MM (Fig. 2).

Subsequently, in both groups, standard ACLR was performed using closed fixed-loop suspensory fixation for a quadruple hamstring autograft on the femoral side. The anatomical ACL femoral footprint was located, and the central area between the AM and posterolateral bundle attachments of the ACL was marked as the target site for femoral tunnel drilling. The femoral tunnel intra-articular aperture was marked before drilling based on the anatomical ACL femoral footprint location in both portal groups.

The knee joint was placed in maximal hyperflexion before proceeding with tunnel preparation. The femoral tunnel guide pin was then drilled precisely over the premarked site through a group-based medial portal using a zero-offset guide. For patients with ≤ 28 mm tunnel lengths, a switch to the MAIM portal technique was implemented before drilling the tunnel (the crossover patient group). A cutoff of 28 mm was selected to ensure that a minimum of 15–20 mm of the graft would remain within the femoral tunnel, providing at least a 7 mm intact cortical bridge for suspensory fixation support [19, 20]. For these patients, only data obtained before the change in drilling portal technique were considered for analysis. Patients whose short tunnel persisted even after the portal change and those who underwent fixation techniques other than suspensory fixation were excluded. Two investigators, who were blinded to the employed technique, used a measuring scale to determine the distance from the femoral tunnel-drilling portal to the patellar tendon. A measurement was taken from the medial border of the patellar tendon toward the center of the portal, parallel to the joint line, with the knee flexed to 90°. The final measurement was determined as the mean of the two measurements. The interobserver correlation of measurements was assessed using the intraclass correlation coefficient (ICC).

### Outcome measurements

#### Tunnel length

Tunnel length was measured intraoperatively in both groups after drilling the tunnel track using a 4.5 mm drill equipped with a cannulated depth gauge.

#### Tunnel inclination

**Fig. 3:**
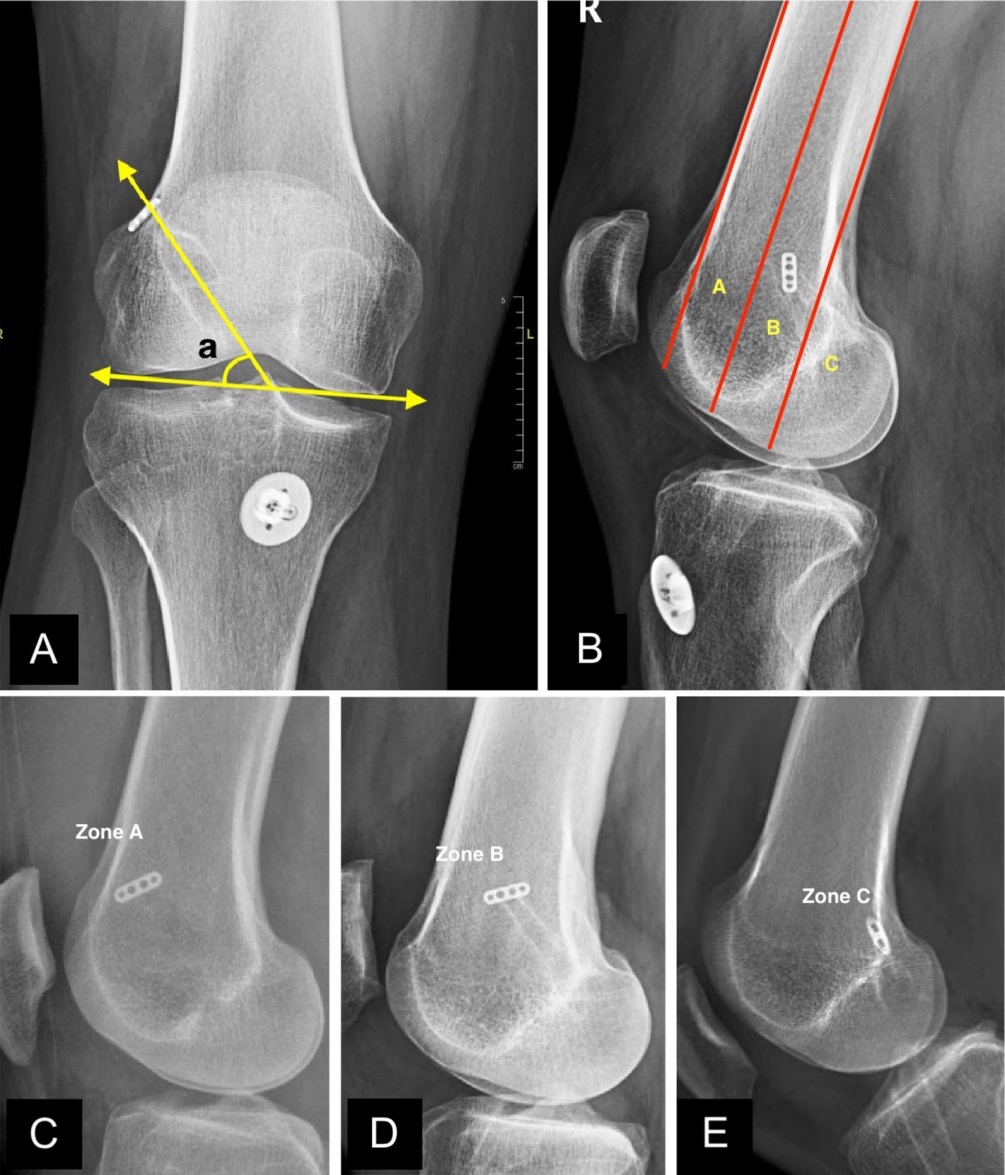
**A** Representative image of femoral tunnel inclination angle “a” measurement in a postoperative AP radiograph of the knee joint following ACL reconstruction; **B** depiction of the categorization of the tunnel exit on lateral radiographs of the knee joint into zones **A**, **B**, and **C**; with examples (**C**–**E**) illustrating zones **A**, **B**, and **C**. ACL, anterior cruciate ligament; AP, anteroposterior

In postoperative true AP radiographs of the knee joint, with the patella positioned centrally over the femoral condyles (Fig. 3A), two trained and independent investigators, who were blinded to the femoral tunnel drilling technique employed, measured the acute angle between the femoral tunnel axis—the central line through the visible tunnel extent—and a line connecting the farthest distal articular extents of the femoral condyles [21]. The mean of their measurements was used, and the interobserver correlation of these measurements was assessed using the ICC.

#### Tunnel exit

**Fig. 4:**
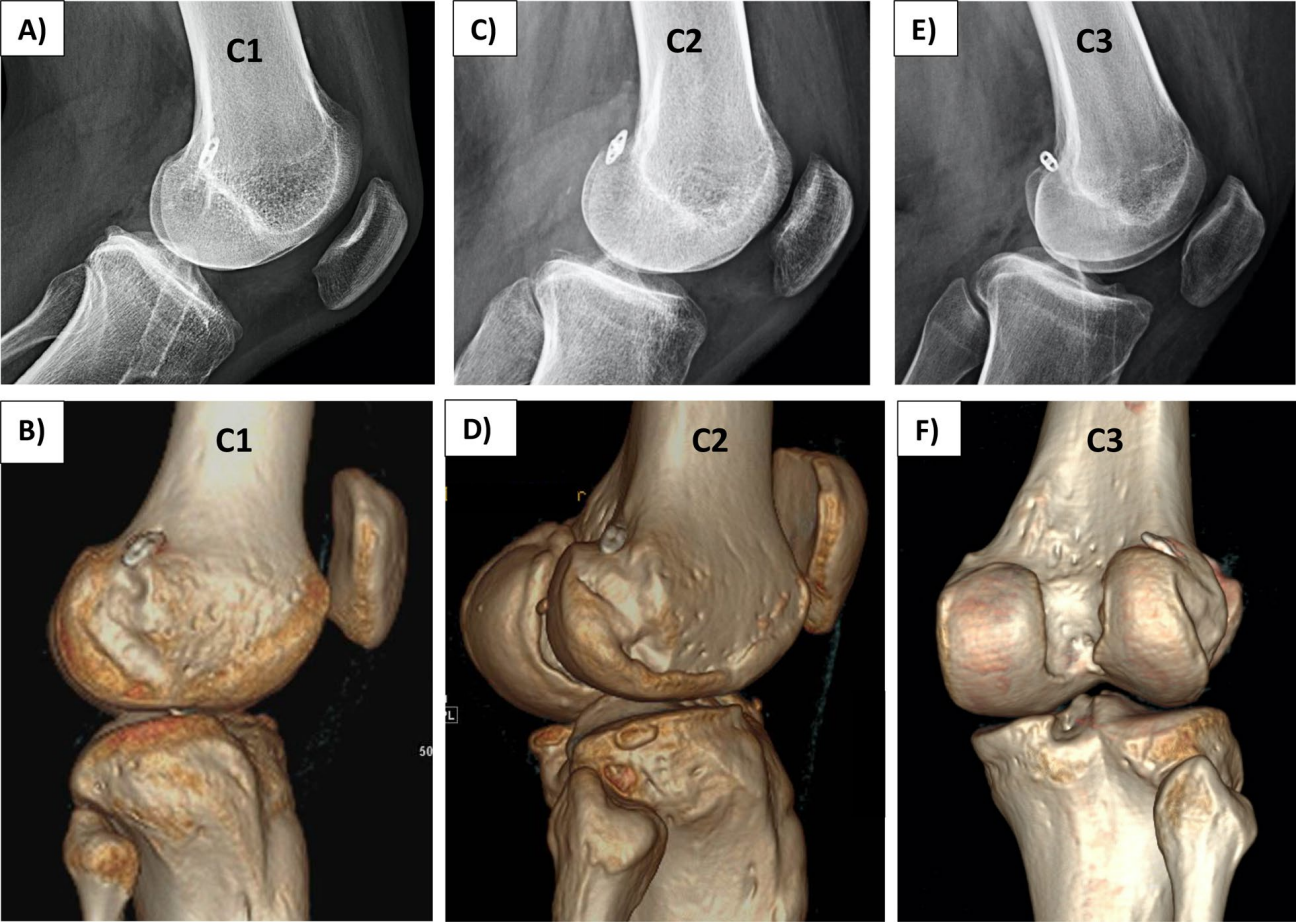
Representative image (X-ray and CT) of femoral tunnel exit type C. Zone C was subdivided into three subzones based on tunnel exist location: 1-lateral (**A** and **B**), 2-posterolateral (**C** and **D**), and 3-posterior (**E** and **F**)

In true lateral knee radiographs (Fig. 3B), where the posterior aspects of the medial and lateral femoral condyles are superimposed, the distal femur was divided into three zones from anterior to posterior. They include the following: (a) the anterior zone was located anterior to the central axis of the visible diaphysis; (b) the middle zone was positioned posterior to the central diaphyseal axis but anterior to the distal extrapolation of the posterior diaphyseal cortical line; and (c) the posterior zone was situated posterior to the distal extrapolation of the posterior diaphyseal cortical line, encompassing the lateral condylar posterior extent. The posterior tunnel exit carries a risk of tunnel blowout owing to the remaining posterior bone thickness, which is limited [9]. Figures 3C–E show the zone of the suspensory fixation device. To provide a more detailed characterization of the anterior and posterior tunnel exits on lateral radiographs, zone C was further subdivided anteroposteriorly into the following three subzones: lateral (C1), posterolateral (C2), and posterior (C3) **(**Fig. 4). While C1 represents the most anterior location, C3 represents the most posterior location, and C2 is located in between. All patients in zone C underwent a computed tomography (CT) scan to precisely determine the exit location of the aperture and confirm the absence of any blowout that was missed intraoperatively. Two independent investigators blinded to the portal technique assessed the tunnel exits on lateral radiographs, and interobserver agreement was analyzed using Cohen’s kappa coefficient (*k*).

#### Complications

The incidence of tunnel blowout, MFC chondral injury (using the Outerbridge classification) [22], and any procedure-related intraoperative complications were recorded. Tunnel blowouts were assessed and categorized arthroscopically into the following: medial blowouts, occurring at the entry aperture of the femoral tunnel [23]; posterior blowouts, occurring within the tunnel owing to posterior bone insufficiency [13, 23]; and lateral blowouts, occurring at the exit aperture of the tunnel [24]. In the case of a lateral tunnel blowout, an extended button was used to salvage the suspensory fixation. All patients were followed up for a minimum of 2 years to record any late tunnel-related complications and instances of ACLR failures, including graft rupture or the need for revision surgery.

The primary outcomes included comparing femoral tunnel length, inclination, and tunnel exit between the MIAM and FAM portal techniques. Secondary objectives encompassed assessing the incidence of MFC injury, tunnel blowout, short tunnel length, posterior tunnel exit, and other complications. They also included investigating the association between patient-related factors such as age, body mass index (BMI), graft diameter, and tunnel length and inclination. The final analysis included 234 patients, with 114 and 120 patients in the MIAM and FAM portal groups, respectively. Three patients in the MIAM group were excluded owing to surgical deferrals, and a further three patients were lost to follow-up. Table 1 presents the baseline demographic characteristics of the patients.

**Table 1:** Demographic parameters of patients included in the MIAM and FAM groups

Demographic parameter	Total (*n* = 234)	MIAM portal (*n* = 114)	FAM portal (*n* = 120)	Statistical comparison
Age (in years)	29.76 ± 6.70 (range: 18–56)	29.15 ± 7.166 (range: 18–51)	30.34 ± 6.18 (range: 19–56)	*p* = 0.09
Female: male distribution	4:230	1:113	3:117	*p* = 0.65
BMI (kg/m^2^)	27.07 ± 4.96 (range: 16.5–44.4)	26.87 ± 4.71 (range: 16.5–40.3)	27.26 ± 5.20 (range: 18.1–44.4)	*p* = 0.45
Right/left	138/96	66/48	72/48	*p* = 0.76
Graft diameter for the femoral tunnel (mm)	7.99 ± 0.84 (range: 7–11)	8.03 ± 0.84 (range: 7–11)	7.96 ± 0.84 (range: 7–11)	*p* = 0.67

*BMI* body mass index, *FAM* far anteromedial, *MIAM* modified inferior anteromedial

### Statistical analysis

Patient demographic parameters, such as age, sex, BMI, and laterality, were recorded. All patient-related quantitative parameters followed a normal distribution as per the Kolmogorov–Smirnov test. Intraoperative tunnel length, graft diameter, tunnel inclination angle, tunnel exit location, and intraoperative complications were documented. Continuous parameters were expressed as the mean ± standard deviation, while categorical parameters were presented as proportions. Student’s *t*-tests were used to compare quantifiable demographic and outcome parameters between the two groups. Fisher’s exact test was used to compare proportion-based measurements. A multiple linear regression analysis was performed to predict the influence of demographic parameters and portal distance from the patellar tendon on tunnel length and inclination in both groups. Additionally, the distributions of these parameters in zone C exits were compared between both portal groups. Finally, tunnel-related parameters were compared between obese (BMI ≥ 30 kg/m^2^) and non-obese (BMI < 30 kg/m^2^) patients within the same and across portal groups. Statistical significance was set at* p*-value ≤ 0.05. IBM SPSS Statistics for Windows (Version 22.0; Armonk, NY: IBM Corp.) software was used for data analysis.

## Results

Patient baseline demographic characteristics were found to be comparable (Table 1). Table 2 presents a detailed comparison of outcome variables between both groups. Significant differences were observed between the two groups regarding tunnel-related parameters. The interobserver ICCs for measuring femoral tunnel inclination and the portal distance from the patellar tendon were both markedly high (0.89 and 0.92, respectively). An excellent agreement (*k* = 1) was observed in predicting tunnel exit locations on plain radiographs. In the FAM group, two patients experienced blowouts (one in the C2 zone and the other in the C3 zone). Among the ten patients in the FAM group with MFC chondral injury, three were classified as Outerbridge grade I, and seven were categorized as Outerbridge grade II. All the MFC chondral injuries were in the non-weight-bearing lateral zone of the MFC. No instances of medial or posterior blowout or MM injuries were observed. In the FAM group, six patients had shorter tunnel measurements ranging from 22 to 26 mm. However, the crossover of these patients to the MIAM portal resulted in larger tunnels, with lengths ranging from 38 to 42 mm. These crossover patients were not included in the MIAM group analysis. Additionally, no patients had a persistently short tunnel length, even after crossover; therefore, no patients underwent a change in the fixation technique other than suspensory fixation.

**Table 2:** Comparison of different outcome variables between the two portal groups

Variable	MIAM portal	FAM portal	Statistical significance
Portal distance from the patellar tendon (mm)	10.07 ± 0.95 (range: 8–12)	34.38 ± 3.54 (range: 28–42)	*p* < 0.05
Femoral tunnel length (mm)	42.43 ± 4.36 (range: 33–55)	31.51 ± 2.69 (range: 22–38)	*p* < 0.05
Femoral tunnel inclination (°)	44.06 ± 6.24 (range: 34–61)	38.43 ± 5.77 (range: 22.4–49)	*p* < 0.05
Tunnel exit zone	A: 12 B: 96 C: 9	A: 0 B: 38 C: 76	*p* < 0.05
Subgrouping of zone C tunnel exit	C1: 9 C2: 0 C3: 0	C1: 46 C2: 23 C3: 7	*p* < 0.05
Tunnel-related complications (*n*)	Nil	Lateral blowout: *n* = 2 MFC chondral injury: *n* = 10 Intraoperative button subsidence: *n* = 2	*p* < 0.05
Number (%) of patients with a short tunnel (< 28 mm)	None	6 (5%)	*p* < 0.05
Significant factors influencing tunnel length (regression coefficient)	BMI (−0.38)	BMI (−0.08)	*p* < 0.05 for specified factors
Significant factors influencing tunnel inclination (regression coefficient)	BMI (−0.43)	1. BMI (−0.24) 2. Graft diameter (1.45) 3. Portal medialization distance (−0.77)	*p* < 0.05 for specified factors

*BMI* body mass index, *FAM* far anteromedial, *MIAM* modified inferior anteromedial

Significantly higher BMI and tunnel lengths were observed in zone C tunnel exits in the MIAM group than in the FAM group (Table 3). However, tunnel inclination measurements were comparable.

**Table 3:** A comparison of tunnel parameters in zone C tunnel exits between the two groups

Variables	MIAM portal	FAM portal	Statistical significance
BMI (kg/m^2^)	35.31 (range: 31.8–40.3)	28.03 (range: 19.7–44.4)	*p* < 0.05
Tunnel length (mm)	34.66 (range: 33–36)	30.77 (range: 28–33)	*p* < 0.05
Tunnel inclination angle (°)	35.83 (range: 34.5–38.5)	36.50 (range: 22.5–47.5)	*p* = 0.70

*BMI* body mass index, *FAM* far anteromedial, *MIAM* modified inferior anteromedial

A comparison of tunnel parameters between patients who were obese and those who were not indicated that non-obese patients had longer tunnels, higher inclination angles, and no posterior exiting tunnels in both groups (Table 4). In the MIAM and FAM portal groups, obese patients exhibited significantly shorter femoral tunnel lengths and significantly lesser inclination angles on AP radiographs than did non-obese patients. When comparing the two portal groups, obese patients in the MIAM portal group had significantly longer femoral tunnels and a higher inclination on AP radiographs than did obese patients in the FAM group. In addition, both obese and non-obese patients in the MIAM group exhibited longer tunnels and higher inclination angles than did their counterparts in the FAM group. Posterior tunnel exits (zone C) were significantly more frequent in obese and non-obese patients in the FAM group than in their counterparts in the MIAM group. Posterior tunnel exits were observed exclusively in obese patients and not in non-obese patients in the MIAM group. However, no significant differences were observed in the occurrence of posterior tunnel exits between obese and non-obese patients in the FAM group.

**Table 4:** A comparison of tunnel parameters in obese and non-obese patients within the same and different portal groups

Variable	Obese versus non-obese patients in the MIAM group	Obese versus non-obese patients in the FAM group	Obese patients in the MIAM group versus obese patients in the FAM group	Non-obese patients in the MIAM group versus non-obese patients in the FAM group
Tunnel length (mm)	39.50 (range: 33–48) versus 43.38 (range: 37–55)*	30.31 (range: 24–35) versus 32.01 (range: 22–38)*	39.50 (range: 33–48) versus 30.31 (range: 24–35)*	43.38 (range: 37–55) versus 32.01 (range: 22–38)*
Tunnel inclination (°)	41.37 (range: 34.5–58) versus 44.95 (range: 34–61)*	36.26 (range:22.5–49) versus 39.61 (range: 25–48)*	41.37 (range: 34.5–58) versus 36.26 (range:22.5–49)*	44.95 (range: 34–61) versus 39.61 (range: 25–48)*
Proportion of zone C tunnel exits (%)	32.14 versus zero*	77.14 versus 57.64	32.14 versus 77.14*	Zero versus 57.64*

*FAM* far anteromedial, *MIAM* modified inferior anteromedial

^*^Indicates statistically significant comparisons (*p* < 0.05)

The mean follow-up period was 36.5 ± 8.3 months (range, 25–46 months). Only one failure was observed in the MIAM portal group, which was a traumatic rupture of the ACL graft at 8 months postoperatively owing to a contact injury while playing soccer. In contrast, two failures occurred in the FAM portal group. One patient experienced a traumatic rupture of the ACL graft owing to a non-contact soccer injury at 7 months postoperatively, while another suffered a traumatic rupture of the ACL graft while returning to sports activity (soccer) prematurely at 4 months postoperatively. The failure rates were comparable between the two portal groups (*p* > 0.05). All other patients had returned to pre-injury activities at the last follow-up visit.

## Discussion

This study revealed that the choice of portal location for femoral tunnel drilling had a significant effect on femoral tunnel length, inclination, and lateral exit location. Furthermore, the findings underscored the superiority of the MIAM portal over the FAM portal for femoral tunnel drilling, resulting in improved tunnel length, exit location, and protection against MFC injury. The utilization of this technique resulted in a longer oblique tunnel that exited more anteriorly on the lateral cortex without the potential risk of posterior wall blowout, lateral cortex breach, or tunnel shortness. This study aimed to provide greater clarity regarding the preferred approach for portal creation based on anatomical single-bundle ACLR. However, the clinical implications of the improvement in tunnel parameters remain unknown.

**Fig. 5:**
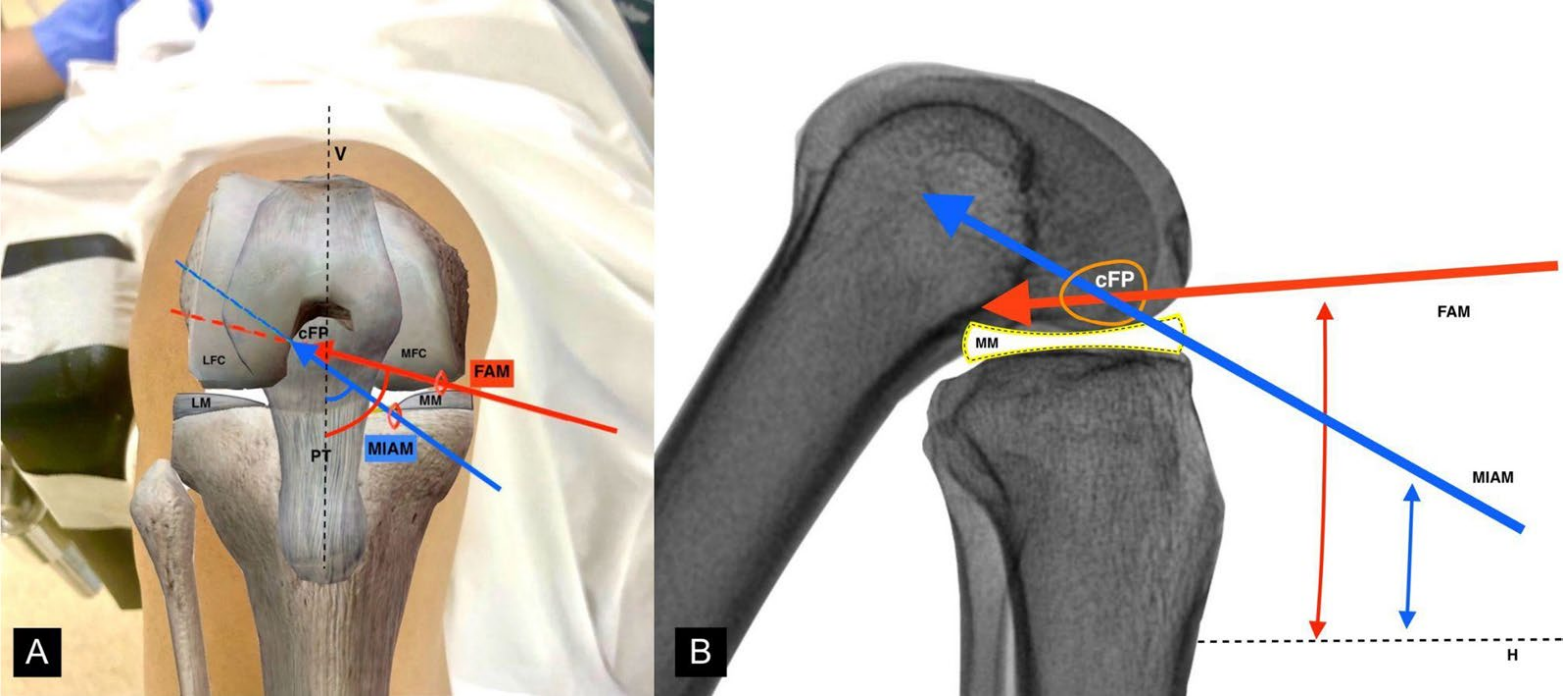
Illustrations showing the effect of portal location on axial inclination and sagittal angulation. Representational images showing the effect of change in axial orientation (**A**) relative to a vertical line “V” and sagittal angulation (**B**) in relation to a horizontal line “H” on the femoral tunnel trajectories, with the two portals in knee hyperflexion. cFP, center of footprint of anterior cruciate ligament; FAM, far anteromedial portal; LFC, lateral femoral condyle; LM, lateral meniscus; MFC, medial femoral condyle; MIAM, modified inferior anteromedial portal; MM, medial meniscus; PT, patellar tendon

The MIAM portal offered a less oblique route for femoral tunnel drilling in the axial plane (in relation to a vertical line passing through the intercondylar notch) than did the FAM portal technique (Fig. 5A). Iyyampillai et al. [25] defined axial inclination as an oblique drilling direction in the axial plane when the knee is in hyperflexion. However, the axial inclination was not investigated in this study, which actually requires a three-dimensional assessment. Rather, this study was focused on the radiographic measurement of the inclination angle in the AP view. Nonetheless, the axial inclination has been discussed to understand the potential basis of longer tunnels and nonposterior tunnel exits with the MIAM portal based on published literature. A lower axial inclination is associated with a reduced risk of a posterior tunnel exit. A better tunnel length resulting from an oblique tunnel trajectory correlates to a lesser axial inclination [25], consistent with the findings in this study. In contrast, the FAM portal exhibits a higher axial inclination, necessary for orthogonal drilling to the lateral condylar medial wall, resulting in a short tunnel and a potential risk of posterior-tunnel exit [25]. Another important modification performed in the MIAM portal was the added scope for inferior positioning because of the meniscus-free zone, which allows for better control in directing the drill towards the anterior tunnel exit and away from the posterior cortex (Fig. 5b). This phenomenon has been termed sagittal inclination, denoting the angle between the femoral shaft axis and the inclination of the tunnel in the sagittal plane [25]. When the knee joint is in a hyperflexed position, it allows a higher sagittal inclination angle than that obtained in a mid-flexion position, where the presence of the meniscus hinders the inferior toggling of the drill. This results in a smaller sagittal inclination. Basdekis et al. [26] reported that the knee flexion angle influenced the position of femoral drilling. A 110° knee flexion angle was found to be the optimum angle, while 90° was associated with a short tunnel that was closer to the posterior wall, increasing the risk of posterior wall blowout. However, these findings correlated with the standard AM portal [26]. In the modified portal utilized in this study, which compensates for meniscus hindrance, longer tunnel lengths were achieved even in patients with obesity, where achieving a hyperflexed knee joint position can be challenging.

The angular data from previous studies involving either of the two portal techniques are less useful for drawing comparative conclusions because of the absence of control groups [27]. The femoral tunnel inclination in the AP view was slightly more horizontal with the FAM portal in this present study, and the tunnel inclination values for both techniques were sufficiently oblique to ensure a nonvertical tunnel, which is different from those of the transtibial route [1, 25]. A higher femoral tunnel inclination angle was correlated with longer tunnels in both groups. The FAM portal clearly results in an overly horizontal tunnel, whereas an MIAM portal-created tunnel is neither horizontal nor vertical. Moreover, the MIAM portal offers the advantage of longer tunnel lengths. The tunnel inclination angle for anatomical ACLR has been reported to range from 29.3° to 57.4°, and the values for both portals in this study fell well within this range [28]. This suggests that rotational stability is likely to be maintained with both portal techniques. From a biomechanical perspective, a proximal tunnel exit is preferable to a lower tunnel exit when considering the load to failure [29]. The thicker cortex is located approximately 3 cm above the lateral epicondyle, which aligns with the stable positioning of the suspensory button [29].

**Fig. 6:**
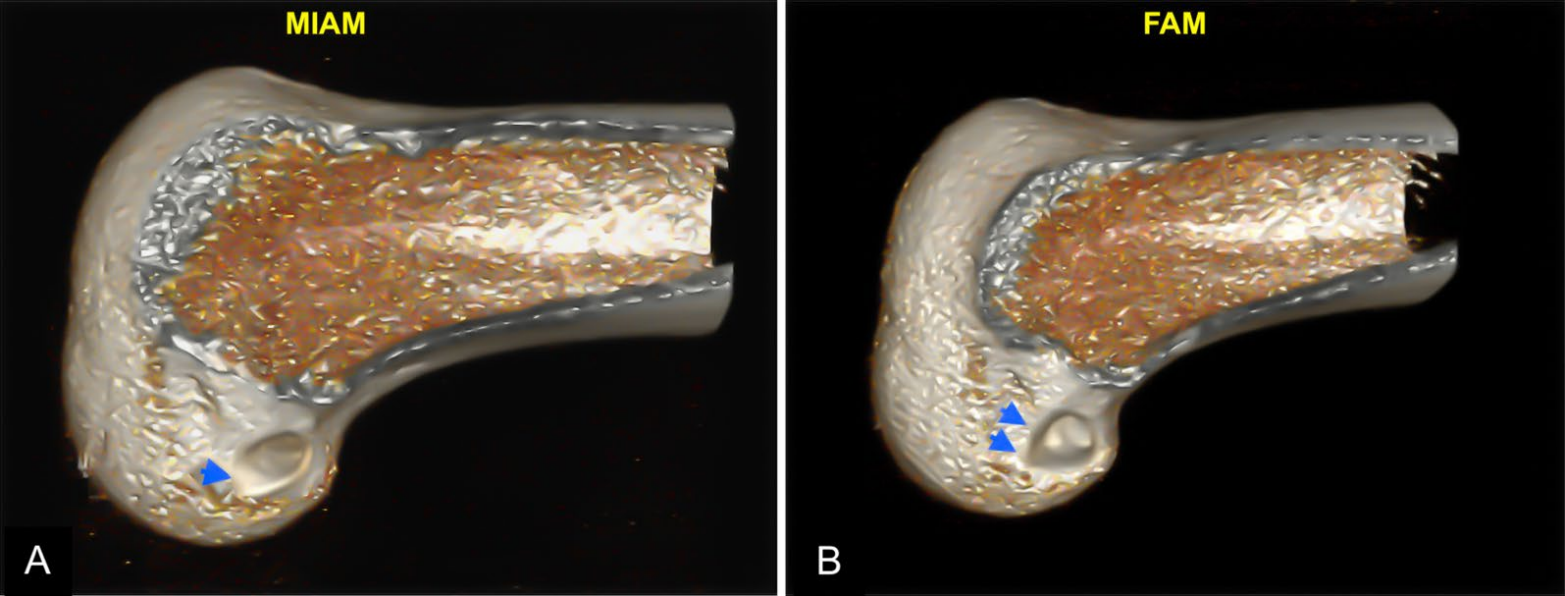
Three-dimensional CT images of entry apertures in the femoral tunnels. **A** The oval entry aperture of the femoral tunnel created through the MIAM portal (single arrowhead), and **B** a near-round entry aperture of the femoral tunnel created via the FAM portal (double arrowheads). CT, computed tomography; FAM, far anteromedial portal; MIAM, modified inferior anteromedial portal

Regarding the entry aperture of the femoral tunnel, the entry aperture assumes a more oval shape when drilled at a steep angle to the notch, such as in transtibial drilling [11, 30]. A perfectly round aperture is only achieved when the drill enters perpendicular to the notch wall, which can be challenging with TP techniques [11, 30]. In the case of the MIAM portal, the entry point was positioned 1 cm medial to the patellar tendon, reducing the likelihood of the drilling track assuming a sharp angle, as might occur with a central portal. However, the difference in tunnel inclination between the two portals was < 6°. This may have contributed to the oval shape of the entry aperture in the MIAM portal for the femoral tunnel. Figure 6 shows that even the aperture of the FAM portal was not perfectly round, whereas that of the MIAM portal was clearly oval. However, it remains unclear how the presence of an oval-shaped entry might contribute to ACLR outcomes, although there is evidence suggesting that oval entry apertures could promote better graft-bone healing [31]. Given that no tunnel-related complications were observed during the follow-up period, it suggests that a minor alteration in the entry aperture may not be of clinical significance. However, a comprehensive analysis of tunnel entry aperture shape and its effect on ACLR outcomes was beyond the scope of this current study; therefore, further studies are required.

Some complications related to lateral wall blowout and suspensory fixation subsidence were observed with the FAM portal, while no such complications were reported with the MIAM portal. However, the posterior and more horizontal placement did carry the risk of lateral wall blowout and potential injury to local structures, respectively [9]. As for ACL graft ruptures, they are rare, occurring because of sports-related injuries, with one rupture resulting from a premature return to sporting activities. An examination of the graft failure results showed that all three patients with graft rupture had a small diameter of 7 mm, which may have contributed to these incidents [32].

In cases where closed fixed-loop suspensory fixation is the preferred method for femoral graft fixation in ACL, the tunnel length is a crucial factor. Generally, a graft-inset length of 20 mm within the femoral tunnel is recommended [33]. However, to ensure that the suspensory button remains properly seated without sinking into the bone, an ideal tunnel length > 35 mm has been suggested [25]. The optimum graft length inside the tunnel for optimal osteointegration remains undetermined. The choice of setting the cutoff for graft inset within the femoral tunnel at a range between 15 and 20 mm was influenced by research conducted by Guglielmetti et al. [19]. Their study demonstrated a higher incidence of residual laxity in patients with a tunnel graft length ≤ 2 cm than in those with a graft length > 2 cm. Furthermore, Mariscalco et al. [34] found that graft lengths as short as 15 mm within the tunnel could be used without leading to adverse consequences.

Previous studies have suggested a wide variation in tunnel lengths due to varying AM portal locations [1, 6, 34]. In this study, the femoral tunnel length was sufficient, with a mean measurement of > 42 mm when created using the MIAM portal technique. However, the tunnel length was significantly shorter when using the FAM portal technique. The mean measurement was < 32 mm and, upon further investigation, only 11% of patients had a femoral tunnel length ≥ 35 mm.

Another challenge associated with low-exiting tunnels is the difficulty in performing additional ligamentous reconstructions in patients with multiligamentous injuries [35]. The posterior half of the lateral surface of the lateral femoral condyle has limited space for tunnel creation in multiligamentous injuries, especially in cases involving the posterolateral and AL ligaments. An inaccurate tunnel creation can result in tunnel convergence, potentially carrying the risk of reconstruction graft damage, damage to fixation devices, and poor graft fixation, leading to reconstruction failure [35].

Many studies have assessed the AM portal and its modifications for femoral tunnel creation; however, only a few studies advocate the use of an AM portal adjacent to or near the patellar tendon [7, 9, 36].

MFC chondral injury was observed in ten patients from the FAM portal group, while none were in the MIAM portal group. The injuries were classified as low grade (Outerbridge grades I and II), and the weight-bearing surface was not involved. Upon further investigation, all patients with chondral injuries had a graft diameter > 9 mm, suggesting that the use of a larger drill diameter may potentially play a role in these MFC chondral injuries. However, no cases of MFC chondral injury were observed in the MIAM portal group. This may be attributed to a relatively less oblique entry in the intercondylar condylar notch, resulting from less medialization. The MIAM portal is located approximately 1 cm medial to the standard anterior portal, which is located adjacent to the patellar tendon. However, the MIAM portal is not at an extreme medial location; rather, it follows a standardized medialization approach by maintaining the entry point approximately 1 cm medial to the medial edge of the patellar tendon. In contrast, the lack of a standardized medialization technique in the FAM portal increases the risk of MFC injury. Therefore, the suitability of the FAM portal may not be ideal and would depend on the extent of medialization required. Conversely, the MIAM portal, with its standardized medialization approach, potentially reduces the risk of MFC injury.

BMI emerged as the major factor influencing tunnel morphology in both groups. The negative association between obesity (higher BMI) and tunnel length observed in this study is likely attributed to obesity-related limitations in achieving knee hyperflexion in both groups [25, 37]. However, the tunnel length was longer in patients who were obese and non-obese in the MIAM group, showing an approximate 1 cm difference. This implies the potential advantage of using the MIAM portal technique to circumvent the limitations associated with knee hyperflexion.

While the findings in this study support the utilization of the modified inferior anteromedial portal as opposed to the far anteromedial portal, the experience of the surgeon and anatomical variation may influence the outcome of the technique. Hence, a more quantifiable method should be utilized to make the result more reproducible for other surgeons. Therefore, arthroscopic guidance was relied on during the creation of the MIAM portal in this study. The ideal portal location should be at the meniscus-free zone area (located just lateral to the anterior horn of the medial meniscus) and as inferior as possible, above the tibia plateau rim. In this study, this location was found to be approximately 1 cm medial to the patella tendon. However, portal location may vary between patients, implying that the 1 cm medialization may not be universally applicable, especially in cases with larger or smaller knees or varying degrees of subcutaneous fat thicknesses. Therefore, relying solely on the skill of the surgeon or specific anatomical landmarks (such as the distance from the patella tendon) for the creation of the MIAM portal can pose challenges of reproducibility for other surgeons, while arthroscopic guidance during portal creation will help overcome these challenges.

This study had some limitations. First, the design was not randomized. Utilizing a blinded randomized controlled trial would potentially reduce bias and allow for a more robust investigation into the causal association between portal location and long-term radiological and clinical outcomes. Second, the clinical outcomes of the two portal techniques were not analyzed in detail, especially the functional outcomes. However, the available information regarding ACLR revision/failure rates provided sufficient data indicating that no major tunnel-related complications occurred during the > 2 year follow-up period. Third, the findings do not account for individual morphological variations in the knee anatomy, including bony structures, which could affect tunnel morphology. Therefore, more sophisticated analyses are required to address this concern. Fourth, determining the influence of the degree of knee flexion, instrumentation, and footprint location was beyond the scope of this study, all of which may have been confounding factors. Hence, further research to consider these factors is warranted. Finally, this study focused exclusively on the prospective analysis of intraoperative tunnel morphology using two portal techniques. A randomized controlled trial would likely contribute to advancing understanding regarding how these techniques affect long-term radiological and clinical outcomes.

## Conclusions

The MIAM portal allowed for better control in relation to tunnel length, inclination, and nonposterior femoral tunnel exit than that obtained with the FAM portal. The MIAM portal was more beneficial for obese patients with shorter tunnels, who face a greater risk of a posterior exit when the FAM portal is used.

## Data Availability

The data may be provided upon request to the corresponding author.

## Abbreviations

ACL: Anterior cruciate ligament
ACLR: Anterior cruciate ligament reconstruction
AL: Anterolateral
AM: Anteromedial
AP: Anteroposterior
BMI: Body mass index
CT: Computed tomography
FAM: Far anteromedial
ICC: Intraclass correlation coefficient
*k*: Cohen’s kappa coefficient
MFC: Medial femoral condyle
MIAM: Modified inferior anteromedial
MM: Medial meniscus
TP: Transportal
